# Epithelioid Fibrous Histiocytoma (EFH) With Rare PPFIBP1-ALK Fusion: A Predominantly Spindle Cell Variant Within the Emerging Spectrum of Myxoid Spindle Cell EFH

**DOI:** 10.7759/cureus.86361

**Published:** 2025-06-19

**Authors:** Eli P Oldham, Svetlana Bobkova, Patti Loykasek, Jennifer Roberts, Igor Shendrik

**Affiliations:** 1 Office of Medical Student Research, Oklahoma State University Center for Health Sciences, Tulsa, USA; 2 National Center for Wellness and Recovery, Oklahoma State University Center for Health Sciences, Tulsa, USA; 3 Molecular, Immunohistochemistry, and Flow Cytometry, Pathology Laboratory Associates, Tulsa, USA; 4 Dermatology, Jennifer Roberts M.D. Dermatology, Norman, USA; 5 Dermatopathology, Regional Medical Laboratory, Tulsa, USA; 6 Dermatopathology, Pathology Laboratory Associates, Tulsa, USA

**Keywords:** alk gene fusion, anaplastic lymphoma kinase, epithelioid cell histiocytoma, epithelioid fibrous histiocytoma, sams

## Abstract

Epithelioid fibrous histiocytoma (EFH) is a benign fibrohistiocytic neoplasm characterized by morphologic heterogeneity and recurrent anaplastic lymphoma kinase (*ALK*) gene rearrangements. We present a rare case of EFH located on the lateral neck of a 56-year-old male patient, demonstrating a predominantly spindle cell morphology. Immunohistochemical analysis revealed granular cytoplasmic positivity for ALK, along with expression of CD68, CD4, epithelial membrane antigen, caldesmon, and smooth muscle actin. Next-generation sequencing confirmed the presence of a rare *PPFIBP1-ALK* fusion. The presented case highlights a predominantly spindle cell variant of EFH and suggests inclusion within the recently described myxoid spindle cell EFH spectrum, which encompasses the superficial ALK-rearranged myxoid spindle cell neoplasms (SAMS).

## Introduction

Epithelioid fibrous histiocytoma (EFH) is a benign fibrohistiocytic neoplasm, which was first described as a distinctive cutaneous neoplasm in 1989 by Jones et al., under the name “epithelioid cell histiocytoma” [1,2]. Initially considered a morphologic variant of benign fibrous histiocytoma (BFH), recent molecular and histological insights have demonstrated that EFH is a biologically distinct entity. EFH commonly presents as a well-circumscribed, polypoid dermal lesion with an epidermal collarette, and predominantly affects the lower extremities of adults; however, it has also been documented in other anatomical sites [3,4].

A hallmark of EFH is its association with recurrent rearrangements of the anaplastic lymphoma kinase (*ALK*) gene. These rearrangements, first reported in EFH by Doyle et al. in 2015, serve as a critical diagnostic tool [5]. It was subsequently demonstrated that EFH may present with a morphologic spectrum that may complicate differentiation from other dermal neoplasms, including BFH, dermatofibrosarcoma protuberans (DFSP), and inflammatory myofibroblastic tumors (IMT) [2,6].

Our case presents a rare example of EFH with a *PPFIBP1-ALK* fusion, a genetic alteration that has only been reported once previously in the literature [4]. The lesion demonstrated unusual histologic features, prompting immunohistochemical (IHC) and molecular testing to support definitive interpretation.

## Case presentation

**Figure 1:**
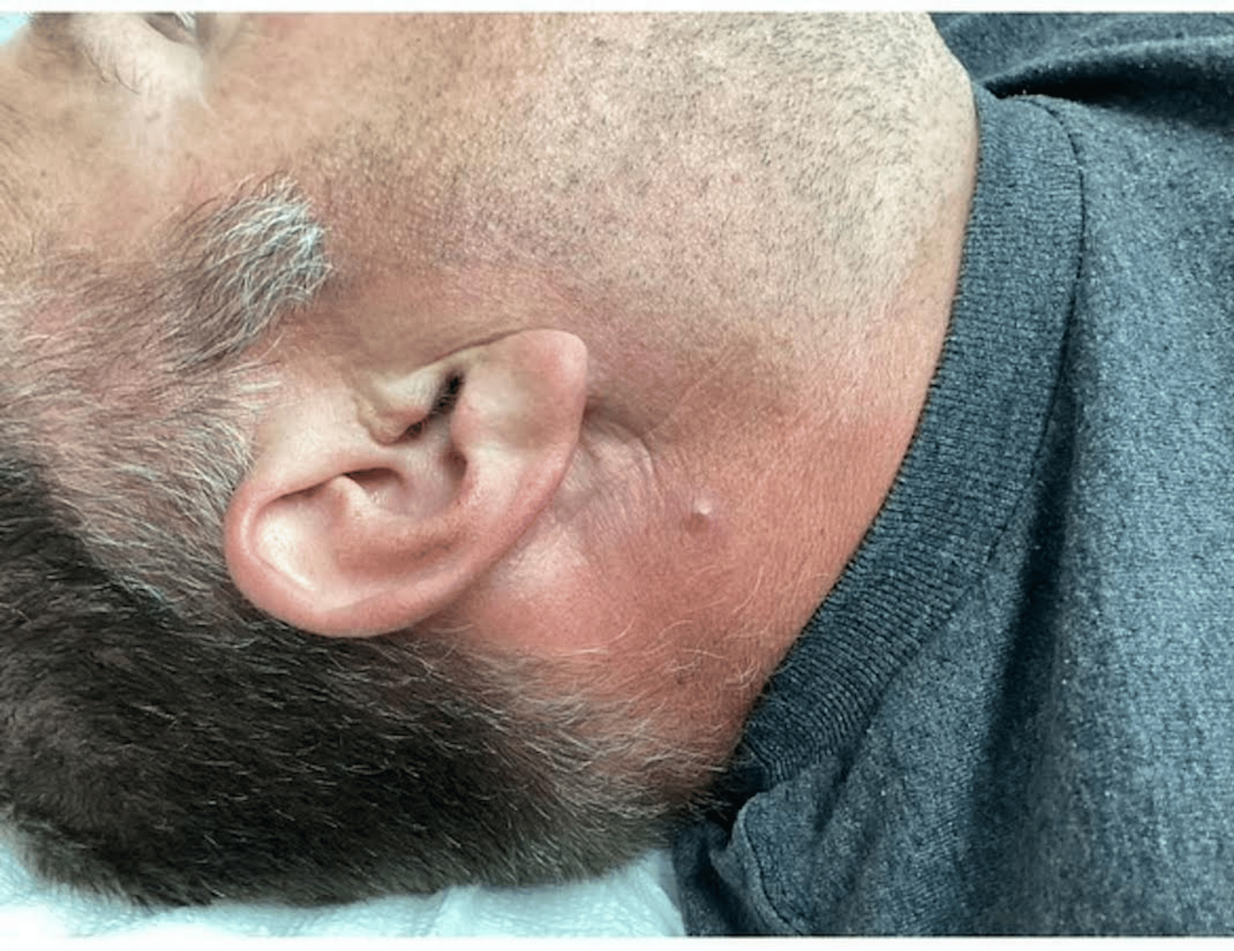
Clinical presentation showing a solitary, dome-shaped, firm papule on the lateral neck region.

A 56-year-old male patient presented with a solitary, asymptomatic lesion on the right lateral neck, just inferior to the ear and adjacent to the angle of the mandible (Figure 1). The lesion had been present for approximately six months, gradually increasing in size but causing no discomfort. Clinically, the differential diagnosis included dermatofibroma, basal cell carcinoma, Spitz nevus, and benign adnexal tumors.

Physical examination revealed a well-defined, dome-shaped papule measuring approximately 5 mm in diameter. The lesion had a smooth surface with a slightly erythematous base and mild surrounding erythema. On palpation, the papule was firm, tender, and slightly indurated. There were no signs of ulceration, scaling, active drainage, or satellite lesions. The adjacent skin did not show significant inflammation or pigmentary alterations.

**Figure 2:**
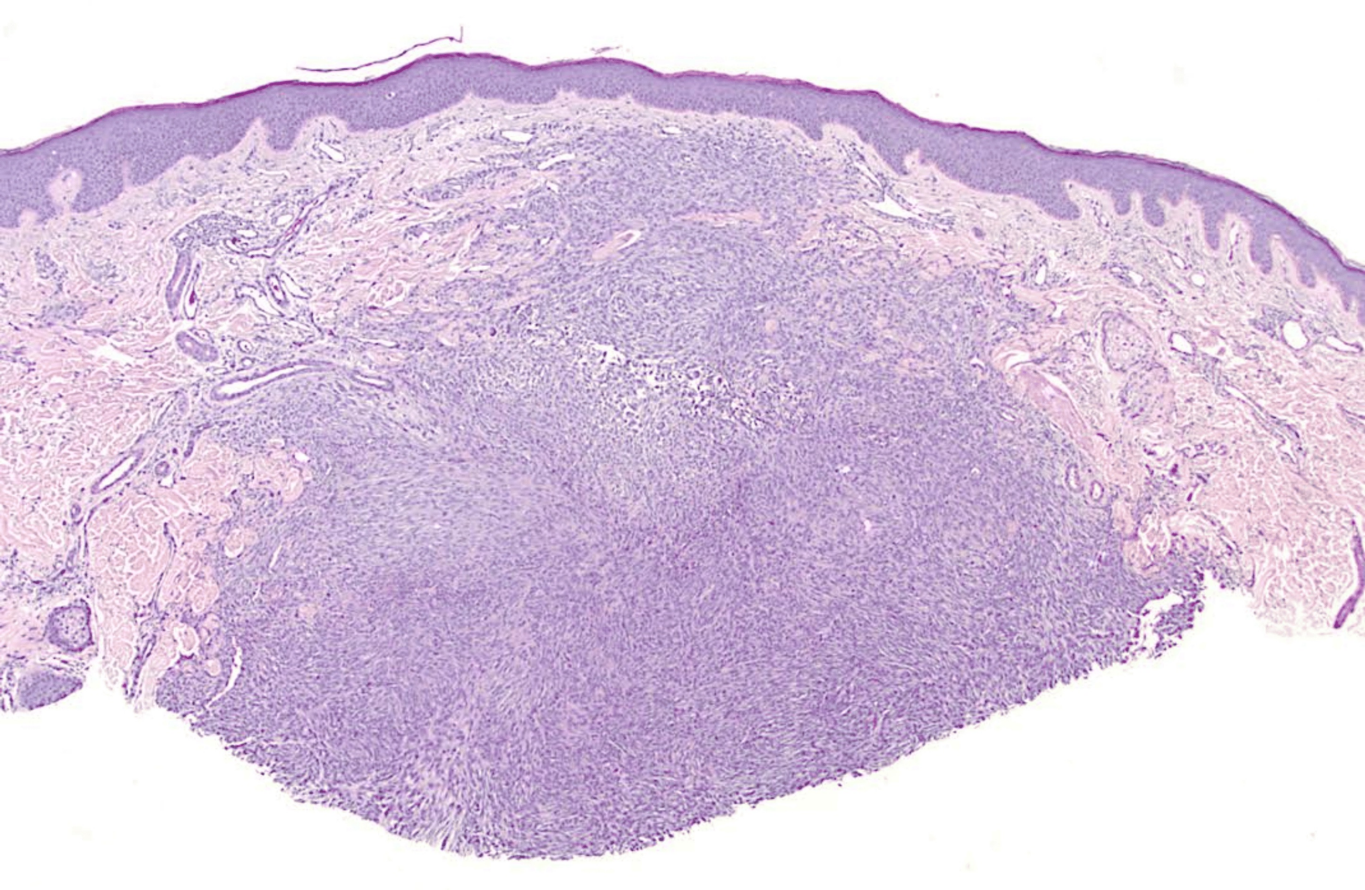
Low-power histologic view demonstrating a sharply circumscribed dermal lesion beneath minimally hyperplastic epidermis (H&E x20).

**Figure 3:**
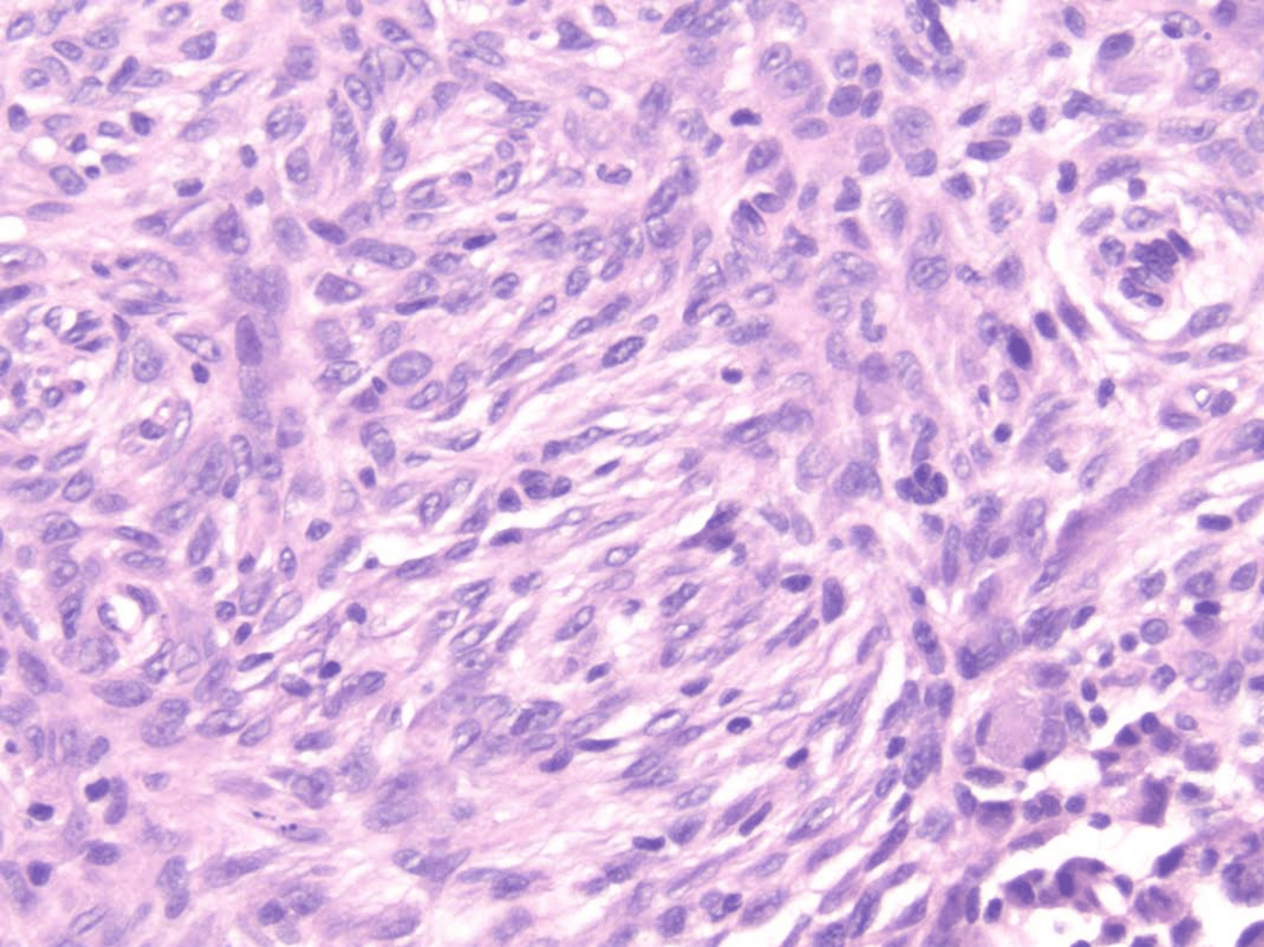
Higher magnification revealing predominantly spindle-shaped cells arranged in sheets and fascicles with focal epithelioid morphology (H&E x200).

A biopsy was performed, demonstrating a sharply circumscribed dermal proliferation beneath an intact epidermis with minimal hyperplasia (Figure 2). The tumor was primarily located within the superficial to mid dermis, characterized by cellular nodules composed predominantly of spindle-shaped and epithelioid cells arranged in sheets, with focal fascicular architecture (Figure 3). While the tumor demonstrated variable cellularity, no apparent myxoid areas were present. The lesional cells exhibited abundant eosinophilic cytoplasm, round to oval nuclei with open chromatin, and small inconspicuous nucleoli. Mitotic figures were infrequent, and there was no evidence of significant nuclear pleomorphism or necrosis. Occasional multinucleated giant cells with granular cytoplasm were noted. Sparse hemosiderin deposits were identified in deeper portions of the lesion. Scattered lymphocytes and small blood vessels were interspersed throughout the tumor. The stroma showed variable collagen deposition, with a more delicate fibrous network evident at the periphery of the lesion. No excessive stromal hyalinization, ectatic vessels, or amianthoid collagen was seen. The nature of the biopsy precluded evaluation of the deep surgical margin and relation of the tumor to the subcutaneous tissue.

**Figure 4:**
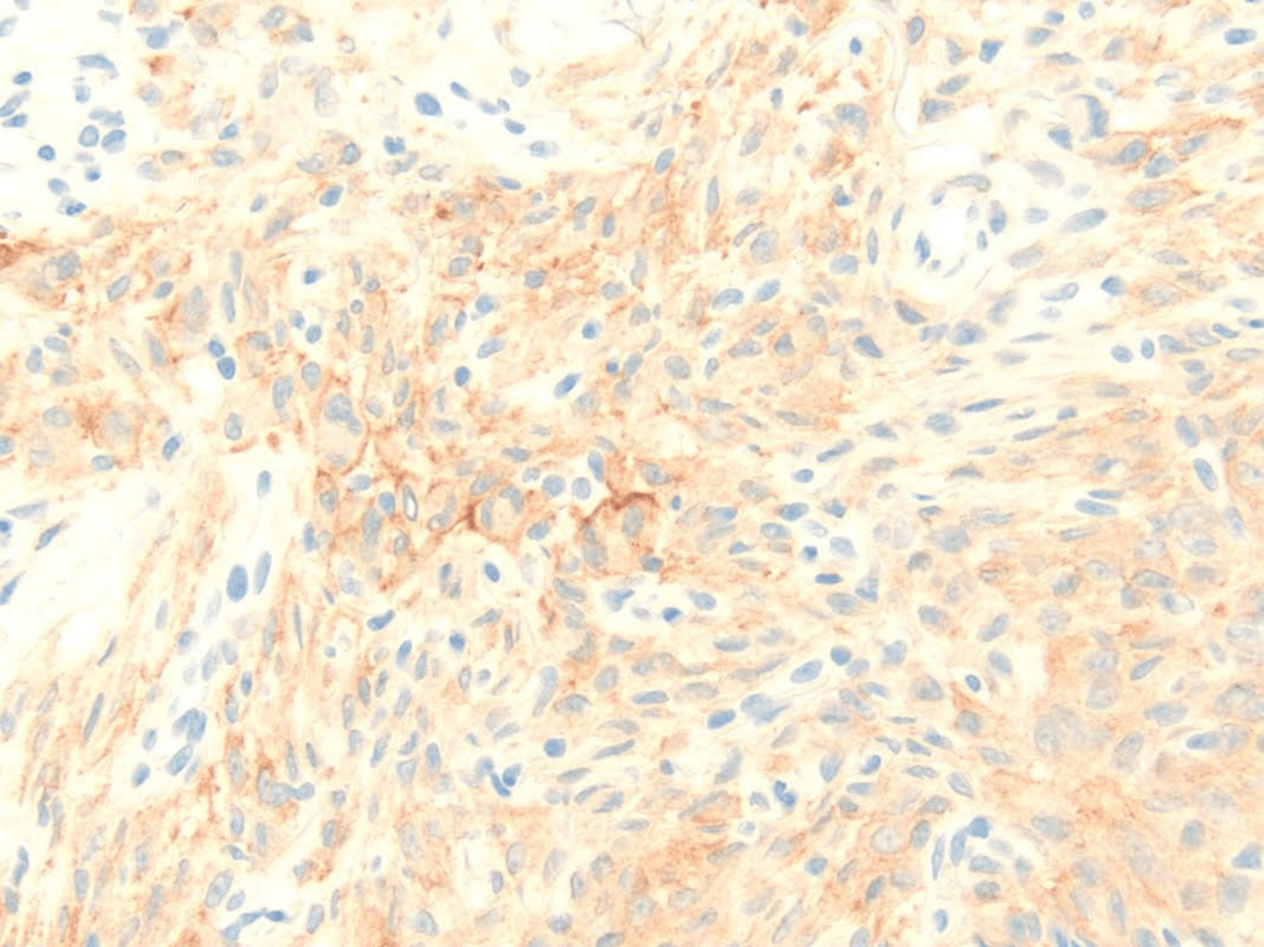
Immunohistochemistry demonstrating diffuse granular cytoplasmic positivity for ALK, supporting the diagnosis of EFH (ALK x200). EFH: epithelioid fibrous histiocytoma

IHC studies demonstrated granular cytoplasmic positivity for ALK, confirming ALK protein overexpression (Figure 4). Additionally, the tumor cells expressed CD68 diffusely, consistent with their histiocytic nature. CD4 staining highlighted approximately 50% of the tumor cells. epithelial membrane antigen (EMA), caldesmon, and smooth muscle actin (SMA) staining were weakly positive, observed only focally. Factor XIIIa and CD163 highlighted scattered cells. S100 protein positivity was limited to isolated dendritic cells. There was no expression of CD34, CD1a, desmin, or CD30, effectively excluding endothelial, myogenic, dendritic cell, and lymphomatous proliferations from the differential.

Molecular analysis by next-generation sequencing (NGS) using an RNA-based fusion detection assay revealed the presence of an *ALK* gene rearrangement characterized by an in-frame fusion between the *PPFIBP1 *gene on chromosome 12 and *ALK* gene on chromosome 2, specifically designated as *PPFIBP1-ALK* (fusion transcript *PPFIBP1-ALK.P9A20*), with the genomic breakpoint precisely located between exon 9 of *PPFIBP1* and exon 20 of *ALK* (chr12: *PPFIBP1*; chr2: *ALK*). This fusion confirmed the diagnosis of EFH, a distinctive variant of fibrous histiocytoma associated with *ALK* rearrangement. The lesion was transected at the base. At the eight-month follow-up, there was no clinical evidence of recurrence or additional lesions at the site.

## Discussion

Originally described by Jones et al. in 1989, the lesion of EFH is now defined by a combination of morphologic features, ALK immunoreactivity, and molecular data [1,2].

EFH typically presents morphologically as a polypoid or dome-shaped dermal lesion, frequently accompanied by a characteristic epidermal collarette [3,4]. Morphologic presentations vary considerably; classic EFH exhibits epithelioid cells arranged in sheets and nests, often accompanied by histiocyte-like multinucleated giant cells [1,2]. Variants may also include spindle-cell predominant morphology, lesions with marked cellularity, or lesions with abundant stromal sclerosis, lesions with storiform patterns, and chondroblastoma-like variants [7], complicating differentiation from other dermal neoplasms such as DFSP, IMT, Spitz nevus, or dermatofibroma [2,6].

Genetically, EFH is consistently associated with *ALK* rearrangements. Multiple fusion partners of *ALK* have been identified in EFH, including *SQSTM1-ALK*, *VCL-ALK*, *DCTN1-ALK*, *ETV6-ALK*, *SPECC1L-ALK*, and the rare *PPFIBP1-ALK* fusion [4]. The specific *PPFIBP1-ALK* fusion detected in our case (fusion transcript *PPFIBP1-ALK.P9A20*, chr12:27809663 - chr2:29446394) has been previously reported only once [4].

IHC staining for ALK protein is an important diagnostic marker for EFH. While ALK expression in EFH may vary depending on the test method, it is frequently positive, with rates varying from 76% to 88% of cases, typically manifesting as diffuse cytoplasmic staining with a granular pattern, although membranous or nuclear staining has also been reported in some rare cases [5,8]. The expression of ALK strongly correlates with the underlying *ALK* genetic rearrangements [5]. EFH typically shows diffuse positivity for histiocytic markers such as CD68 and CD163, and variably Factor XIIIa positive, confirming its histiocytic differentiation, but this feature alone is nonspecific [5,7,8]. SMA, caldesmon, and EMA often exhibit weak, focal positivity, but this is also nonspecific and of limited diagnostic utility. Classic EFH consistently lacks significant expression of CD34, desmin, CD30, CD1a, or S100 markers, which helps distinguish it from morphologically similar tumors [5,7,8].

The link between morphologic variability and *ALK *fusion status has been explored, but remains unclear. While certain morphologic patterns, such as perinuclear dot-like staining, may indicate specific fusions like *PRKAR2A-ALK*, no consistent correlation between fusion type and histological features has been established [6,9]. Nevertheless, the identification of *ALK* fusions and expression patterns remains a cornerstone for distinguishing EFH from its histologic mimics [3,5].

*ALK* rearrangements occur in an emerging group of receptor tyrosine kinase (RTK) fusion-positive mesenchymal neoplasms. One recently described superficial tumor entity falling under this category is "superficial ALK-rearranged myxoid spindle cell neoplasm" (SAMS), initially introduced by Dermawan et al. [10]. SAMS is histologically characterized by myxoid whorls of spindled neoplastic cells, consistently expressing ALK, CD34, and frequently S-100 protein by IHC [10].

Recently, a designation of myxoid spindle cell variant of EFH (MS-EFH) was proposed, encompassing cutaneous neoplasms with *ALK* rearrangements exhibiting a broad morphological spectrum from spindle-shaped to predominantly myxoid tumors [11]. MS-EFH demonstrates substantial clinicopathologic overlap with classic EFH, including a predilection for extremities (particularly the lower limb; 71%), frequent epidermal collarettes (63%), and benign clinical behavior without metastatic potential despite frequent involvement of surgical margins [11]. Histologically, MS-EFH is characterized by whorled spindle cell morphology set within a myxoid-to-collagenous stroma, frequently expressing CD34 (71%), EMA (61%), and S100 (28%) [11]. Molecular analyses reveal *ALK* fusions in a majority of cases (79%; 15/19 sequenced cases), with recurrent fusion partners including *SQSTM1 *and *VCL*, alongside novel *RET* and *NTRK3 *fusions identified in a subset (17%; 3/18) [11].

Some described lesions exhibit combined histologic features of both classic EFH (epithelioid cells with eosinophilic cytoplasm) and SAMS-like regions (myxoid spindle cell whorls), with abrupt transitions and distinctive IHC staining profiles [11]. This suggests that SAMS does not represent a separate diagnostic entity but rather constitutes a distinct histologic pattern within the broader MS-EFH spectrum [11].

Recent studies have also revealed that at least a subset of lesions previously categorized as dermal non-neural granular cell tumors may represent variants of EFH, further broadening its morphologic and molecular landscape [12].

Our case demonstrated a predominantly spindle-shaped cellular proliferation, significantly differing from the epithelioid morphology seen in classic EFH. Although epithelioid cells were present, they were not a dominant cellular component, limiting definitive histologic diagnosis. This unusual morphologic presentation necessitated additional IHC and molecular analyses. The identification of granular cytoplasmic ALK immunopositivity strongly supported a diagnosis of EFH, which was confirmed by NGS, revealing the rare *PPFIBP1-ALK *fusion. The molecular identification of this rare fusion transcript confirmed the diagnosis.

Differential diagnosis of EFH principally encompasses Spitz nevus, dermatofibroma, IMT, and DFSP [2,6]. Spitz nevi demonstrate strong S100 and Melan A positivity while lacking *ALK* rearrangement [2]. Dermatofibroma typically exhibits Factor XIIIa positivity but lacks ALK expression [2]. IMT commonly shows prominent inflammatory infiltrates and stronger SMA positivity, often accompanied by more diffuse ALK staining [2]. DFSP characteristically expresses strong CD34 positivity and lacks histiocytic markers and* ALK* rearrangements, allowing definitive differentiation from EFH through IHC and molecular profiling [2].

## Conclusions

A predominantly spindle cell variant of EFH is described. Diagnosis was confirmed by IHC and NGS, the latter identifying a rare *PPFIBP1-ALK* fusion transcript. This lesion's morphologic and molecular features suggest its inclusion within the recently proposed MS-EFH spectrum, which may now incorporate SAMS.
